# Effect of LncRNA XIST on Immune Cells of Primary Biliary Cholangitis

**DOI:** 10.3389/fimmu.2022.816433

**Published:** 2022-03-04

**Authors:** Chunhui She, Yifei Yang, Bo Zang, Yuan Yao, Qixuan Liu, Patrick S. C. Leung, Bin Liu

**Affiliations:** ^1^ Department of Rheumatology, Affiliated Hospital of Qingdao University, Qingdao, China; ^2^ Epidemiology and Biostatistics, Maternal and Child Health, School of Public Health (SPH) Department, Boston University, Boston, MA, United States; ^3^ Division of Rheumatology, Allergy and Clinical Immunology, University of California at Davis, Davis, CA, United States

**Keywords:** Primary biliary cholangitis, LncRNA XIST, CD4^+^ T cells, NKs, Th1, Th17

## Abstract

**Objective:**

Primary biliary cholangitis (PBC) is an autoimmune disease with significant gender difference. X chromosome inactivation (XCI) plays important roles in susceptibility to diseases between genders. This work focuses on the differences of LncRNA XIST in several defined immune cells populations as well as its effects on naive CD4+ T cells proliferation and differentiation in patients with PBC.

**Methods:**

NKs, B cells, CD4+ T, and CD8+ T cells were separated by MicroBeads from peripheral blood mononuclear cells (PBMCs) of PBC patients and healthy control (HC). The expression levels of LncRNA XIST in these immune cells were quantified by qRT-PCR and their subcellular localized analyzed by FISH. Lentivirus were used to interfere the expression of LncRNA XIST, and CCK8 was used to detect the proliferation of naive CD4+ T cells in PBC patients. Finally, naive CD4+ T cells were co-cultured with the bile duct epithelial cells (BECs), and the effects of LncRNA XIST on the typing of naive CD4+ T cells and related cytokines were determined by qRT-PCR and ELISA.

**Results:**

The expression levels of LncRNA XIST in NKs and CD4+ T cells in PBC patients were significantly higher than those in HC, and were primarily located at the nucleus. LncRNA XIST could promote the proliferation of naive CD4+ T cells. When naive CD4+ T cells were co-cultured with BECs, the expressions of IFN-γ, IL-17, T-bet and RORγt in naive CD4+ T cells were decreased.

**Conclusion:**

LncRNA XIST was associated with lymphocyte abnormalities in patients with PBC. The high expression of LncRNA XIST could stimulate proliferation and differentiation of naive CD4+ T cells, which might account for the high occurrence of PBC in female.

## 1 Introduction

Primary Biliary Cholangitis (PBC) is a female predominant autoimmune disease characterized by high titer of anti-mitochondrial antibodies (AMAs) and elevated Alkaline phosphatase (ALP) (1). The incidence ratio of male to female is 1:9 (2). X chromosome is the chromosome with the highest density of immune-related genes. In order to balance the immune system, a chromosome will be permanently inactivated randomly in female development (3). The association of X-chromosome inactivation (XCI) and inflammatory diseases implicates its significance in the gender predominance in autoimmunity (4–6). LncRNA XIST as a critical factor for XCI based on its location within the X-inactivation centre (XIC) and its unique expression pattern that is completely female-specific in adult somatic cells (7). LncRNA XIST engages with proteins in a modular and developmentally controlled manner to coordinate chromatin spreading and silencing (8).

Livers of patients with PBC are highly infiltrated with immune cells around the small and medium bile ducts, which the ratio of CD4/CD8 is reported to be as high as 2.45:1 (9). Dysregulation and abnormal proportion of different subtypes of CD4+ T cells could lead to immune disorders and autoimmune diseases (10). Previous studies had found that the imbalance between CD4+ Th1 and CD4+ Th17 cells was the basis of the immune abnormalities and pathological changes of PBC (11, 12). The characteristic cytokines secreted by Th1 cells include Interferon-γ (IFN-γ) and Tumor Necrosis Factor-α (TNF-α) (13). The characteristic cytokines secreted by Th17 cells include IL-17, IL-5 and IL-6, which mediated humoral immune response (14). Abnormal cytokines often led to excessive inflammatory response and abnormal cell typing, triggering autoimmune disorders, which contributed to the occurrence of diseases (15). Previous studies had found that Xist RNA localization at the Xi could be an important factor for maintaining dosage compensation of X-linked genes in T cells, and abnormal XCI maintenance in T cells was a feature of SLE disease (16, 17). However, the relationship of LncRNA XIST and T cells in PBC have not been done before.

LncRNA XIST is of great significance for the study of autoimmune diseases with significant gender differences (18). The in-depth study of LncRNA XIST in PBC will help to further clarify the pathogenesis of PBC.

## 2 Materials and Methods

This study was approved by the ethics committee of the Affiliated Hospital of Qingdao University, and written informed consent forms were obtained (approval number: QYFY WZLL 25571).

### 2.1 Cell Culture

Peripheral blood mononuclear cells (PBMCs) were collected from 20 patients with PBC (PBC group) and 20 healthy persons (healthy control group, HC) all matched for age and gender. NK cells, B cells, CD4+ T cells and CD8+ T cells in PBMCs were separated by MicroBeads (Miltenyi Biotec, Bergisch Gladbach, Germany) following the manufacturer’s protocols (19). Cells were cultured at 37°C, in an atmosphere with 5% CO_2_.

### 2.2 Flow Cytometry (FCM)

NK cells, B cells, CD4+ T cells and CD8+ T cells were collected and labeled for 30 min at 4°C in the dark with the following monoclonal antibodies (mAbs): CD56-PE, CD16-APC, CD19-PE, CD45RA-FITC, CD4-APC, CD8-APC, CD3-PE according to the manufacturer’s instructions. The FACS Canto II instrument (BD Immunocytometry Systems, CA, USA) was used for data acquisition. Data were analyzed with Diva-8 (BD Immunocytometry Systems, CA, USA) and FlowJo (Tree Star, OR, USA) software.

### 2.3 Fluorescence *In Situ* Hybridization (FISH)

The subcellular localization of lncRNA XIST in the immune cells was identified based on the instructions of LncRNA FISH Probe Mix (CY3 labeled probe) with the application of a FISH assay. NK cells, B cells, CD4+ T cells and CD8+ T cells were fixed on the slide by cytocentrifuge. Add LncRNA FISH Probe Mix (Red) following the manufacturer’s protocols. LncRNA XIST probe were purchased from Shanghai GenePharma Co. After drying, cover with coverslip and observe under confocal laser scanning microscope (20).

### 2.4 Quantitative Real-Time Polymerase Chain Reaction (qRT-PCR)

Total RNA was isolated from cells using RNAiso Plus (TaKaRa, Dalian, China). The cDNA was synthesized from 2.5μg RNA using a commercial One-Step PrimeScript RT-PCR Kit (TaKaRa, Dalian, China). qRT-PCR was monitored online using Roche 480 (Roche, USA). The relative gene expression was normalized to GAPDH and calculated using the 2−ΔΔCT method, where CT was the cycle threshold. The primer sequences are shown in **Table 1**.

**Table 1:** Primer sequences.

Primer	Primer Sequences
**GAPDH**	F: GGAGCGAGATCCCTCCAAAAT
	R: GGCTGTTGTCATACTTCTCATGG
**h-IFN-γ**	F: CGCCAGAGTGGTTATCTTTTGA
	R: CGGTAGTGAACCCGTTGATGT
**h-RORγt**	F: GACAGGGCCCCACAGAGA
	R: TTTGTGAGGTGTGGGTCTTCTTT
**IL-4**	F: CACAGAAGGCAGGGAGTGTGT
	R: AGCCTTCGCTTGGGCTTAAT
**h-T-bet**	F: GCCTACCAGAATGCCGAGATT
	R: ATCTCCCCCAAGGAATTGACA
**h-IL-17**	F: CGCGTTTCCATGTCACGTAA
	R: GATGTCTTCCTTTCCTTGAGCATT
**h-GATA-3**	F: CACAGAAGGCAGGGAGTGTGT
	R: AGCCTTCGCTTGGGCTTAAT

### 2.5 CCK-8 Detection

The naive CD4+ T cells were cultured at 37°C for 3 days, and 10 μL/well CCK-8 detection reagents (Solarbio, China) were added to the 96-well culture plate following the manufacturer’s protocols. After incubation for 2 h at 37°C, the OD value at 450 nm was measured.

### 2.6 Expression Interference of LncRNA XIST in CD4+ T cells

#### 2.6.1 siRNA Sequence Screening

Lipofectamine 2000 was mixed with three different siRNA for 20min at room temperature, and then separately added the naive CD4+ T cells culture medium following the manufacturer’s protocols. The siRNA (GenePharma Co, Shanghai, China) interference sequence screened is:

XIST-homo-3639: GCUGACUACCUGAGAUUUATT,
UAAAUCUCAGGUAGUCAGCTT;
XIST-homo-5529: GCUUCUAACUAGCCUGAAUTT,
AUUCAGGCUAGUUAGAAGCTT;
XIST-homo-6167: GCAUGCAUCUUGGACAUUUTT,
AAAUGUCCAAGAUGCAUGCTT.

RNA was extracted from naive CD4+ T cells after interference, and the expression level of LncRNA XIST in RNA level was detected by qRT-PCR to determine the effectiveness.

#### 2.6.2 Cell Transfection

Lentiviral plasmid containing XIST-homo-3639 (oeXIST) and lentiviral empty plasmid (oeNC) were generated. Naive CD4+ T cells of appropriate density were cultured in 24-well plates. The serum-free RPMI 1640 medium was replaced and starved for 2h. Lipofectamine 2000 and Lentivirus (Heyuan Biotechnology, shanghai, China) were diluted, mixed and added into cells medium for further culture. Naive CD4 cells at 5×10^4^ cells/well in 96-well with oeXIST were used as treatment group, and oeNC were used as control group, and then co-cultured with BECs at a rate of 1:1 respectively (21).

### 2.7 Enzyme-Linked Immunosorbent Assay (ELISA)

The protein levels were verified using an ELISA kit (Cloud-Clone Corp, Wuhan, China) to measure change in IFN-γ, IL-4 and IL-17 after naive CD4+ T cells were co-cultured with BECs. Naive CD4+ T cells were co-cultured with BECs in 96-well plates for 3 days. The supernatants were harvested, and the levels of cytokines (IFN-γ, IL-4 and IL-17) in the supernatants were determined using the ELISA kit following the manufacturer’s protocols.

### 2.8 Statistical Analysis

Date was analyzed using GraphPad Prism version 7.0 software. Data were expressed as mean ± standard error of mean and all experiments were repeated at least three times. Group comparisons were performed using an unpaired, two-tailed Student *t* test. Multiple group comparisons were performed through analysis of variance (ANOVA). A two-way ANOVA was used when data with more than one factor were analyzed. *P* values less than 0.05 were considered statistically significant.

## 3 Results

### 3.1 Differential Expression of LncRNA XIST in Immune Cells of PBC Patients

**Figure 1:**
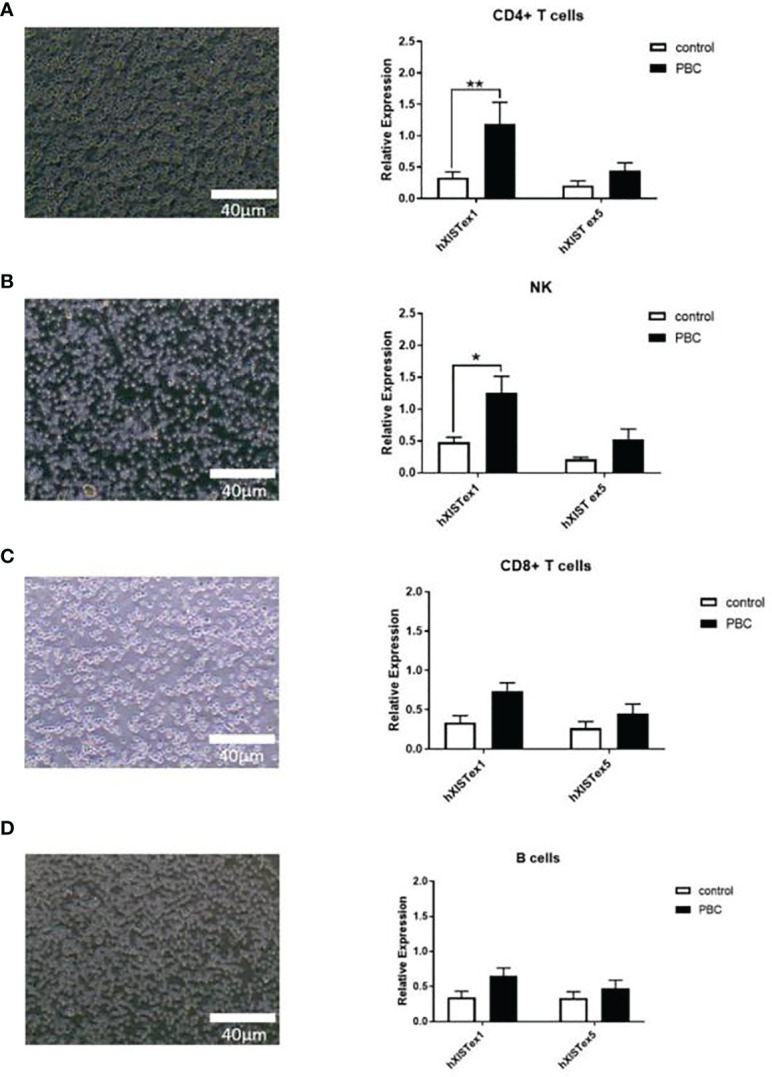
Differential expression of LncRNA XIST in immune cells of PBC patients. Representative images (left, under electron microscope) and qRT-PCR (right) showing expression levels of LncRNA XIST in CD4+ T cells **(A)**, NK cells **(B)**, CD8+ T cells **(C)** and B cells **(D)** from PBC patients (n=20) and healthy controls (n=20). Results presented as mean ± SEM *significantly higher than control. (*p < 0.05; **p < 0.01).

The relevant clinical indicators of PBC patients and HC were shown in **Table 2**. CD4+ T cells, NK cells, CD8+ T cells and B cells were purified from PBMC of patients with PBC and HC by Microbeads (**Figure S1**). There was no significant difference between the immune cells of PBC patients and HC under normal electron microscopy, but there were some differences in the levels of XIST ex1 and XIST ex5 were examined by qRT-PCR. The expression of LncRNA XIST in CD4+ T cells and NK cells were significantly higher in PBC patients than that in HC (1.26 ± 0.09 vs 0.48 ± 0.19, 1.19 ± 1.23 vs 0.34 ± 0.31, p<0.01 and p<0.05). There was no difference in the expression of LncRNA XIST in B cells and CD8+ T cells of PBC patients compared with HC (0.65 ± 0.40 vs 0.35 ± 0.30 and 0.74 ± 0.37 vs 0.34 ± 0.31, p>0.05) (**Figure 1**).

**Table 2:** The relevant clinical indicators of PBC patients and HC.

Index	PBC (n=20)	HC (n=20)	P
Female	100%	100%	
age	59.90 ± 6.24	55.18 ± 5.06	
AMA	100%	0%	
ALT (U/L)	134.24 ± 52.76	19.86 ± 9.43	<0.001
AST (U/L)	63.89 ± 31.23	26.49 ± 12.72	<0.001
TBIL (μmol/L)	89.76 ± 68.51	13.20 ± 3.79	<0.001
ALP (U/L)	160.94 ± 92.90	66.34 ± 19.19	<0.001
IgM (g/L)	1.88 ± 1.30	0.79 ± 0.21	<0.001

AMA, Anti-mitochondrial antibodies; ALT, alanine aminotransferase; AST, aspartate aminotransferase; TBIL, Total bilirubin; ALP, alkaline phosphatase. (^*^P <0.05, ^**^P <0.01, ^***^P <0.001).

### 3.2 The Localization of LncRNA XIST in Immune Cells of PBC Patients

**Figure 2:**
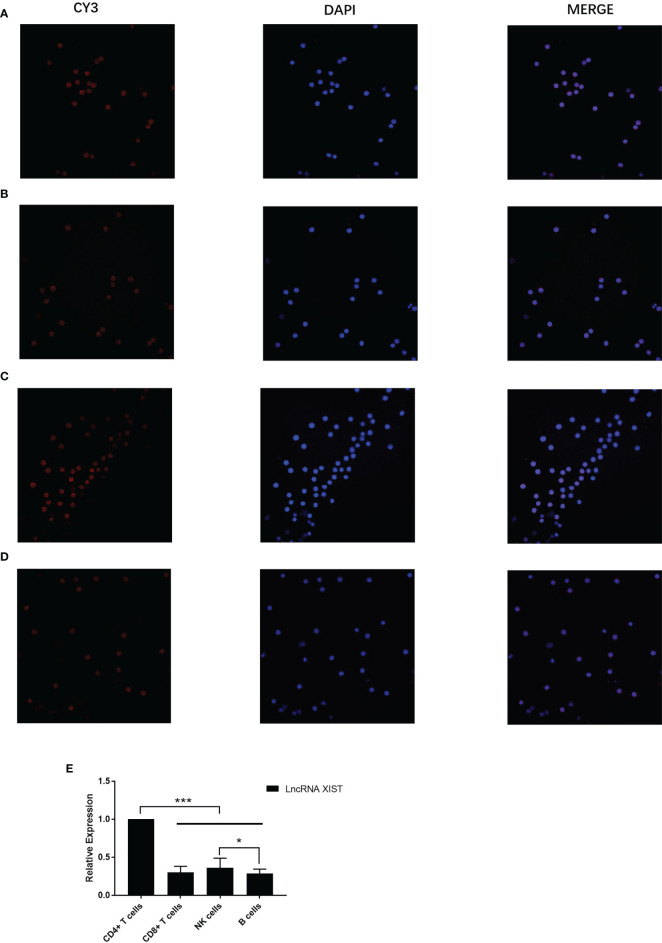
The localization of LncRNA XIST in immune cells of PBC patients were different. Representative images (confocal laser scanning microscope) and the subcellular localization of lncRNA XIST in the immune cells was identified. Red: CY3 labeled LncRNA XIST, blue: DAPI staining. Localization of LncRNA XIST in CD4+ T cells **(A)**, CD8+ T cells **(B)**, NK cells **(C)**, B cells **(D)** of PBC patients (n=20) were different. **(E)** Relative expression of LncRNA XIST in CD4+ T cells, CD8+ T cells, NK cells, B cells. The data are presented as mean ± SEM of three independent experiments (*p < 0.05; ***p < 0.001).

The subcellular localization of lncRNA XIST in the immune cells was identified based on the instructions of LncRNA FISH Probe (CY3 labeled) with the application of a FISH assay. The results showed that LncRNA XIST was clearly located and abundant in CD4+ T nuclei in PBC patients (Red-LncRNA XIST, Blue-DAPI) (**Figures 2A–D**). The expression levels of LncRNA XIST in CD4+ T cells in PBC patients were significantly higher in PBC than that in other groups (1 ± 0 vs 0.37 ± 0.03, 1 ± 0 vs 0.3 ± 0.02, 1 ± 0 vs 0.29 ± 0.01, p<0.005). In addition, the expression levels of LncRNA XIST in NK cells in PBC patients were also higher than that in B cells (0.37 ± 0.03 vs 0.29 ± 0.01, p<0.05) (**Figure 2E**).

### 3.3 Expression Interference of LncRNA XIST in CD4+ T Cells

**Figure 3:**
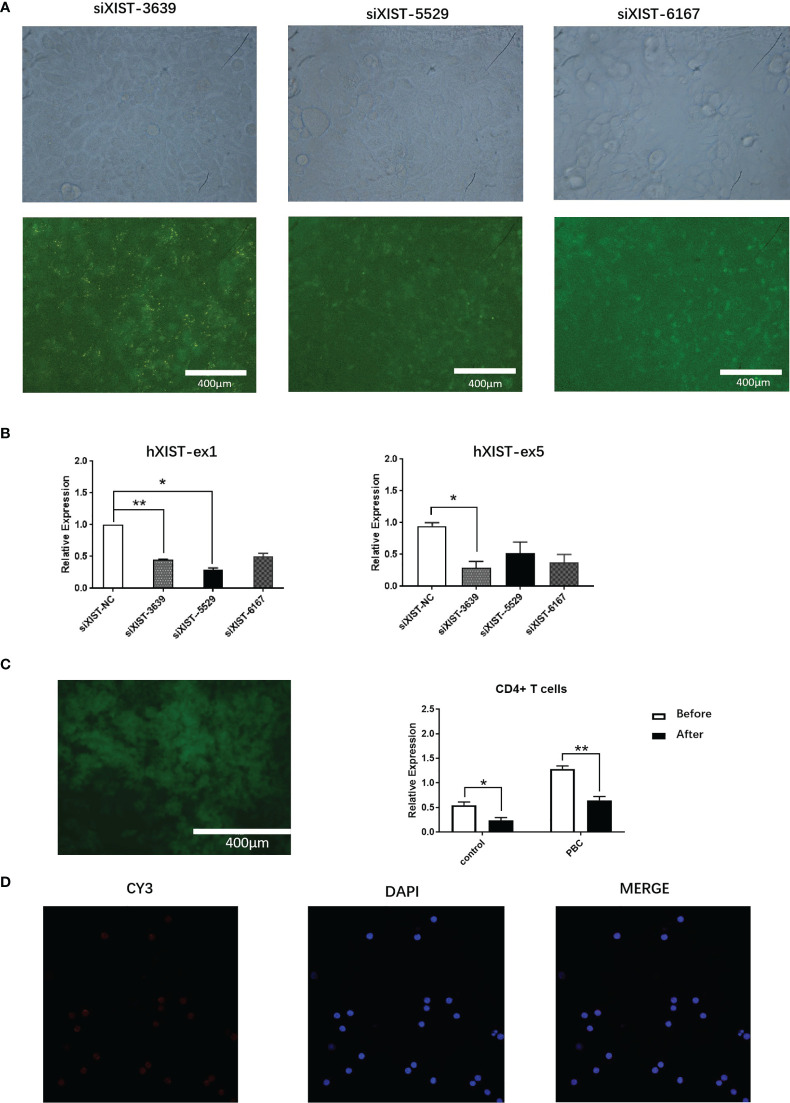
Expression interference of LncRNA XIST in CD4+ T cells of PBC patients. **(A)** Representative images (fluorescent microscope) and naive CD4+ T cells were transfected with siXIST-3639, siXIST-5529, or siXIST-6167, respectively. **(B)** The levels of XIST-ex1 and XIST ex-5 were examined by qRT-PCR in naive CD4+ T cells transfected with siRNA negative control (siXIST-NC), siXIST-3639, siXIST-5529, or siXIST-6167, respectively. The data are presented as mean ± SEM of three independent experiments (n=3 *p < 0.05; **p < 0.01). **(C)** Representative images (fluorescent microscope) and qRT-PCR was used to detect the expression of LncRNA XIST at RNA level after naive CD4+ T cells were transfected with lentiviral oeXIST. The data are presented as mean ± SEM of three independent experiments (n=3, *p < 0.05; **p < 0.01). **(D)** Representative images (confocal laser scanning microscope) and LncRNA XIST is localized in the nucleus of naive CD4+ T cells were transfected with lentiviral oeXIST.

Three distinct siRNA (siXIST-3639, siXIST-5529 and siXIST-6167) were examined for their capacity in interfering LncRNA XIST expression in naive CD4+ T cells (**Figure 3A**). Specifically, siXIST-3639 effectively reduced the expression of LncRNA XIST but not the other two siRNAs tested (**Figure 3B**). To maintain low expression of LncRNA XIST in naive CD4+ T cells. The selected sequences were prepared to interfere with naive CD4+ T cells by lentiviruses. As expected, the expression of LncRNA XIST in naive CD4+ T cells was significantly reduced when naive CD4+ T cells were treated with lentivirus oeXIST-3639(0.55 ± 0.12 vs 0.24 ± 0.11, 1.28 ± 0.11 vs 0.65 ± 0.14, p<0.01) (**Figure 3C**). Using FISH, reduction of the number of LncRNA XIST localized in naive CD4+ T cells were demonstrated (**Figure 3D**).

### 3.4 LncRNA XIST Affects the Proliferation of Naive CD4+ T Cells and Production of IFN-γ, IL-17, T-Bet and RORγt

**Figure 4:**
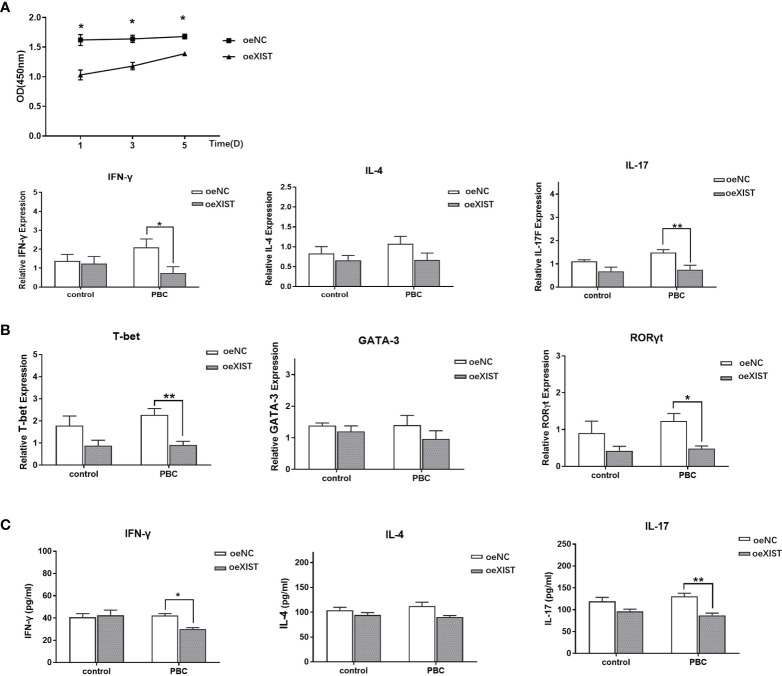
LncRNA XIST affects the proliferation and difference of naive CD4+ T cells. **(A)** The proliferation of naive CD4+ T cells in PBC patients (n=20) was analyzed by CCK8. Compared with naive CD4+ T cells in the oeNC group, the proliferation ability of naive CD4+ T cells in the oeXIST group was decreased at different time periods. The data are presented as mean ± SEM of three independent experiments. *p < 0.05. **(B)** After co-culture, qRT-PCR was used to detect the expression of IFN-γ, IL-4, IL-17, T-bet, GATA-3 and RORγt in PBC patients (n=20). The data are presented as mean ± SEM of three independent experiments (*p < 0.05; **p < 0.01). **(C)** After co-culture, the expression level of IFN-γ, IL-4 and IL-17 at the protein level in PBC patients (n=20) were detected by ELISA. The data are presented as mean ± SEM of three independent experiments (*p < 0.05; **p < 0.01).

The proliferation of naive CD4+ T cells in PBC patients after interference of LncRNA XIST expression by lentivirus was analyzed by CCK8. Compared with naive CD4+ T cells in the oeNC group, the proliferation ability of naive CD4+ T cells in the oeXIST group was decreased at different time periods (p<0.05) (**Figure 4A**).

In order to determine the effect of LncRNA XIST on the differentiation of naive CD4+ T cells, we co-cultured naive CD4+ T cells from PBC patients with BECs for three days. Compared with oeNC group, the expression of IFN-γ, IL-17, T-bet, and RORγt was decreased in oeXIST group in PBC patients (2.10 ± 1.32 vs 0.75 ± 0.99; 1.49 ± 0.36 vs 0.75 ± 0.5; 2.28 ± 0.84 vs 0.91 ± 0.50; and 1.23 ± 0.61 vs 0.48 ± 0.22. p<0.05, p<0.01, p<0.01 and p<0.05), while there was no statistical significance in IL-4 and GATA-3 expression in RNA levels (1.07 ± 0.57 vs 0.67 ± 0.52; 1.40 ± 0.91 vs 0.97 ± 0.73. p>0.05) (**Figure 4B**). The expression of IFN-γ, IL-17and IL-4 were also validated at the protein levels by ELISA. Compared with oeNC controls, the expression levels of IFN-γ and IL-17 was decreased in naive CD4+ T cells of oeXIST group in PBC patients (42.29 ± 3.03 vs 30.13 ± 2.13, p<0.01 and 130.61 ± 12.30 vs 87.00 ± 8. 97, p<0.01). There was no change in IL-4 expression (112.28 ± 13.63 vs 90.34 ± 4.88, p>0.05) (**Figure 4C**).

## 4 Discussion

PBC is female predominant autoimmune disease of the liver mainly characterized by positive AMAs and elevated ALP. The gender difference in PBC is one of the highest among autoimmune disease and the ratio of female to male can be as high as 9:1 (22). Recent studies have suggested that chronic high expression of IFN-γ and the interplay of type I/II interferon could be contributing to the pathogenesis and gender difference in PBC, however the underlying mechanism of such significant gender imbalance in PBC remains an enigma (23–25).

XIST has functions beyond XCI and suggests that XIST can contribute to sex-specific differences underlying inflammatory response by attenuating acute inflammation in women (20). LncRNA XIST silencing protects against sepsis-induced acute liver injury *via* inhibition of the BRD4 expression (26). X-linked gene expression and XIST RNA interactome genes are altered in SLE patient activated B cells (27, 28). Thus, we hypothesized that altered XCI could be a triggering juncture in the pathogenesis of female predominant autoimmune response in PBC.

It is of great significance to explore the pathogenesis of PBC from the direction of the serious imbalance of the male-female incidence ratio (29). In this study, our data showed that LncRNA XIST was correlated with lymphocyte abnormalities in PBC patients. Compared with the HC, the expression of LncRNA XIST in NK cells and CD4+ T cells of PBC patients were higher. Then, lentivirus was used to stably interfere with the expression of LncRNA XIST in naive CD4+ T cells. It was found that the proliferation ability of naive CD4+ T cells was reduced. Compared with the oeNC group, the differentiation of naive CD4+ T cells into Th1 and Th17 was significantly lower in oeXIST group in PBC patients, which suggested that LncRNA XIST was significantly associated with Th1 and Th17 differentiation.

Pathological of PBC patients showed that there was infiltration of immune cells around the small and medium bile ducts in the liver, mainly CD4+ and CD8+ T cells, and the ratio of CD4+ to CD8+ T cells was seriously unbalanced (30). A large number of studies had proved that CD4+ T cells were of great significance in the pathogenesis of PBC (31). Meanwhile, Th1/Th2 imbalance was the immunological basis for the occurrence of PBC (32). Th1, Th2, Th17 and Treg cells were differentiated from naive CD4+ T cells (33). Therefore, the abnormal differentiation of naive CD4+ T cells might be related to the pathogenesis of PBC (34, 35).

As previous studies had shown, abnormal expression of LncRNA XIST could affect XCI, leading to the occurrence of female predominance diseases (36). The effect of LncRNA XIST on naive CD4+ T cells was further explored by interfering the expression of LncRNA XIST in naive CD4+ T cells. CCK8 assay showed that the proliferation ability of naive CD4+ T cells in oeXIST group was reduced, indicating that LncRNA XIST could affect the proliferation of naive CD4+ T cells. This finding is also consistent with the increase of CD4+ T cells in PBC patients and the aggregation phenomenon in BECs (37). Therefore, LncRNA XIST can affect the proliferation of CD4+ T cells.

The study found that the expression of IFN-γ, IL-17, T-bet and RORγt secreted by naive CD4+ T cells in the oeXIST group were lower than in oeNC group. IFN-γ, IL-17, T-bet and RORγt were major factor associated with Th1 and Th17 differentiation. We speculated that LncRNA XIST affected cytokine secretion of naive CD4+ T cells, which might increase the inflammatory response of intrahepatic BECs, thereby contributing to the occurrence of PBC subsequently. The increased content of LncRNA XIST in PBC patients might disturb the inactivation of X chromosome, leading to the escape of immune-related genes on X chromosome, resulting in increased secretion of immune-related cytokines, thus affecting the differentiation of Th1 and Th17. The reciprocal regulation between LncRNA XIST and naive CD4+ T cells might explain, at least in part, the occurrence of female predominance in PBC.

Although LncRNA XIST cannot encode proteins, it may indirectly regulate the expression of target genes by inducing epigenetic modification of genes or acting as miRNA sponge to adsorb miRNA, thus participating in the occurrence of diseases (38). Meanwhile, XCI abnormalities are often accompanied by the escape of immune-related genes (39, 40). For example, on a lupus prone mouse model, the X-linked gene Tlr8 was biallelically expressed in a subset of BMDMs, supporting the hypothesis that inefficient silencing of Tlr8 in females contributes to SLE susceptibility (41). It is necessary to further study which genes in PBC escape to explore the specific mechanism of PBC predisposition in women.

In our study, LncRNA XIST was also expressed in NK cells. However, due to the primary NK cells are difficult to transfect and the culture cycle is short, most NK cells died in the process of co-culture and could not be followed up, so the transfection of NK cells failed. We hope to find a better breakthrough in future NK cell research. It is expected that the study of LncRNA XIST in different immune cells of autoimmune diseases with significant gender differences will be further improved. In addition, since most LncRNA XIST is produced in the nucleus, further investigation is needed to answer the question of how LncRNA XIST functions in PBC and related signaling pathways.

## 5 Conclusions

LncRNA XIST was associated with lymphocyte abnormalities in patients with PBC. The high expression of LncRNA XIST could stimulate proliferation and differentiation of naive CD4+ T cells, which might contribute to the occurrence of PBC. Therefore, this study provides a direction for further exploration of the pathogenesis of PBC.

## Data Availability Statement

The raw data supporting the conclusions of this article will be made available by the authors, without undue reservation.

## Ethics Statement

The studies involving human participants were reviewed and approved by the ethics committee of the Affiliated Hospital of Qingdao University. The patients/participants provided their written informed consent to participate in this study.

## Author Contributions

CS and BL were responsible for the experiment design. YFY and BZ were responsible for the experiment. YY was responsible for the data collation. QL and PL were responsible for the paper writing. All authors contributed to the article and approved the submitted version.

## Funding

This work was supported by National Natural Science Foundation of China (Grant No. 8167060249 and Grant No. 81241094), the Natural Science Foundation of Shandong Province, China (Grant No. ZR2016HM13).

## Conflict of Interest

The authors declare that the research was conducted in the absence of any commercial or financial relationships that could be construed as a potential conflict of interest.

## Publisher’s Note

All claims expressed in this article are solely those of the authors and do not necessarily represent those of their affiliated organizations, or those of the publisher, the editors and the reviewers. Any product that may be evaluated in this article, or claim that may be made by its manufacturer, is not guaranteed or endorsed by the publisher.
