# Supplementary material for: Effect of LncRNA XIST on Immune Cells of Primary Biliary Cholangitis
Source: Front Immunol. 2022 Mar 4;13:816433. doi: 10.3389/fimmu.2022.816433 (PMC8931309; doi:10.3389/fimmu.2022.816433)
Supplement: Supplementary file 1 [file DataSheet_1.pdf]

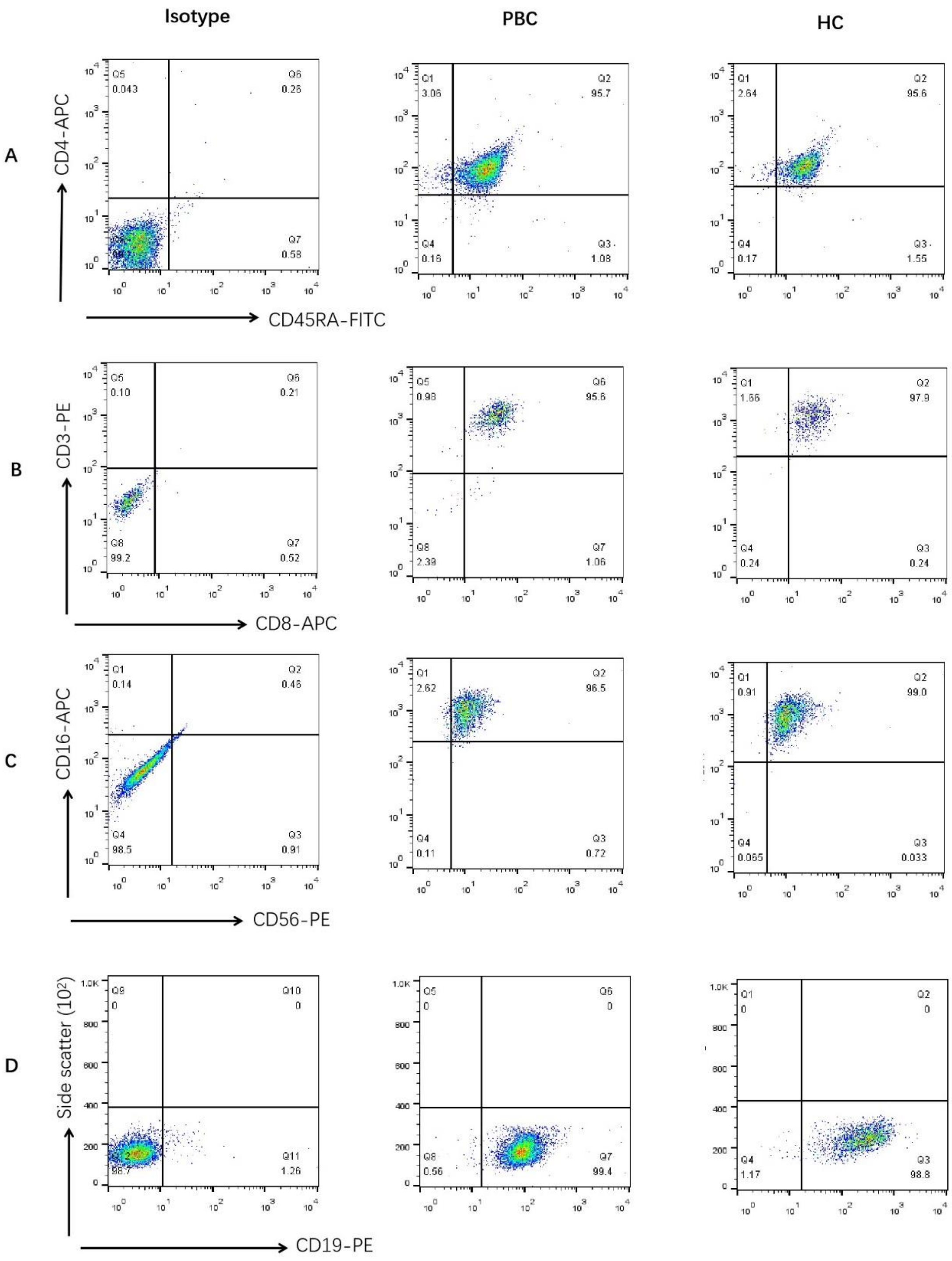

Fig. S1 FACS Analysis of Microbeads purified CD4+ T cells (A), CD8+ T cells (B), NK cells (C) and B cells (D) from patients with PBC and controls. Note that the positive rate of CD4+ T cells, CD8+ T cells, NK cells and B cells in these preparations were all over 95%.
